# Advances in Understanding the Immunological Pathways in Psoriasis

**DOI:** 10.3390/ijms20030739

**Published:** 2019-02-10

**Authors:** Simona-Roxana Georgescu, Mircea Tampa, Constantin Caruntu, Maria-Isabela Sarbu, Cristina-Iulia Mitran, Madalina-Irina Mitran, Clara Matei, Carolina Constantin, Monica Neagu

**Affiliations:** 1 Department of Dermatology, Carol Davila University of Medicine and Pharmacy, 020021 Bucharest, Romania; simonaroxanageorgescu@yahoo.com (S.-R.G.); isabela_sarbu@yahoo.com (M.-I.S.); matei_clara@yahoo.com (C.M.); 2 Department of Dermatology, Victor Babes Hospital of Infectious Diseases, 030303 Bucharest, Romania; 3 Department of Physiology, Carol Davila University of Medicine and Pharmacy, 020021 Bucharest, Romania; 4 Department of Dermatology, Prof. N.C. Paulescu National Institute of Diabetes, Nutrition and Metabolic Diseases, 030167 Bucharest, Romania; 5 Department of Microbiology, Carol Davila University of Medicine and Pharmacy, 020021 Bucharest, Romania; cristina.iulia.mitran@gmail.com (C.-I.M.); madalina.irina.mitran@gmail.com (M.-I.M.); 6 Department of Immunology, Victor Babes National Institute of Pathology, 050096 Bucharest, Romania; caroconstantin@gmail.com (C.C.); neagu.monica@gmail.com (M.N.); 7 Department of Pathology, Colentina University Hospital, 020125 Bucharest, Romania; 8 Faculty of Biology, University of Bucharest, 030018 Bucharest, Romania

**Keywords:** psoriasis, inflammation, immunology, Th-17, IL-17, T regulatory cells

## Abstract

Psoriasis vulgaris is a chronic, immune-mediated, inflammatory, polygenic skin disorder affecting approximately 2% of the population. It has a great impact on quality of life; patients often experience depression, anxiety, stigma as well as suicidal behavior. Even though psoriasis is one of the most studied dermatological conditions, the pathogenesis of the disease is still not completely elucidated. The complex interactions between keratinocytes, dendritic cells, T-lymphocytes, neutrophils and mast cells are responsible for the histopathological changes seen in psoriasis. The pathogenic model leading to the formation of psoriatic plaques has however evolved a lot over the years. There is now enough evidence to support the role of interleukin (IL) -23, IL-17, IL-22, T helper (Th) -17 cells, Th-22 cells, T regulatory cells, transforming growth factor (TGF)-β1 and IL-10 in the pathogenesis of the disease. Moreover, several inflammatory and anti-inflammatory molecules are currently being investigated, some of them showing promising results. The aim of this paper is to look over the most recent advances in the immunological pathways involved in the pathogenesis of psoriasis vulgaris.

## 1. Introduction

Psoriasis vulgaris is a chronic, immune-mediated, inflammatory, polygenic skin disorder affecting approximately 2% of the population. It has a universal occurrence; males and females being equally affected [1,2,3,4]. It can appear at any age, but two peaks in age of onset have been described: the first between 20 and 30 years and the second between 50 and 60 years [4,5]. Plaque-type psoriasis accounts for approximately 90% of cases and clinically manifests as well-demarcated erythematous plaques covered by silvery-white scales with a predilection for the extensor surfaces of the extremities, scalp, sacral area and umbilicus [3,6]. Psoriasis has a great impact on quality of life; patients often experience depression, anxiety, stigma as well as suicidal behavior [7,8].

Even though psoriasis is one of the most studied dermatological conditions, the pathogenesis of the disease is still not completely elucidated [9]. The complex interactions between keratinocytes, dendritic cells (DCs), T-lymphocytes, neutrophils and mast cells are responsible for the histopathological changes seen in psoriasis, namely elongated rete ridges, hyperkeratosis with parakeratosis, Munros’ microabscesses and dilated vessels in the dermal papilla [10,11]. The pathogenic model leading to the formation of the psoriatic plaque has however evolved a lot over the years. While the disease was initially considered an epidermal disorder in which various mediators like cyclic adenosine monophosphate, protein kinase C, phospholipase C, eicosanoids, transforming growth factor (TGF)-α had a central role [6,7], in later years the role of T-cells was recognized and interferon (IFN)-γ and interleukin (IL)-12 were considered key players in the pathogenesis of psoriasis [12]. Tumor necrosis factor (TNF)-α was also intensely studied and the biological therapies targeting this cytokine revolutionized the treatment of psoriasis vulgaris [11,13]. More recently, studies point at the cytokines of the IL-23/IL-17 axis as the important players in the pathogenesis of psoriasis [12]. The aim of this paper is to look over the most recent advances in the immunological pathways involved in the pathogenesis of psoriasis vulgaris.

## 2. Psoriasis Pathogenesis in Brief

Clinical and experimental data sustain the seminal role of the immune system in the pathogenesis of this disease. Even though it is considered a T cell mediated inflammatory pathology, cells that belong to both adaptive and innate immunity as well as non-immune cells are highly involved. Thus, from the first category, dendritic cells, NK cells and macrophages were found as being involved in the pathogenesis of psoriasis, along with cells from the second category; namely keratinocytes and endothelial cells [14]. The autoantigens that activate autoimmune reactions in this disease are still a matter of research. For example, LL-37, an antimicrobial peptide (AMP) that is produced by keratinocytes upon injury, was found overexpressed in moderate to severe psoriatic forms [15]. Another possible auto-antigen could be ADAMTS-like protein 5, produced by injured melanocytes. This auto-antigen can activate the Th17 response, maintaining the psoriatic condition [16].

**Figure 1:**
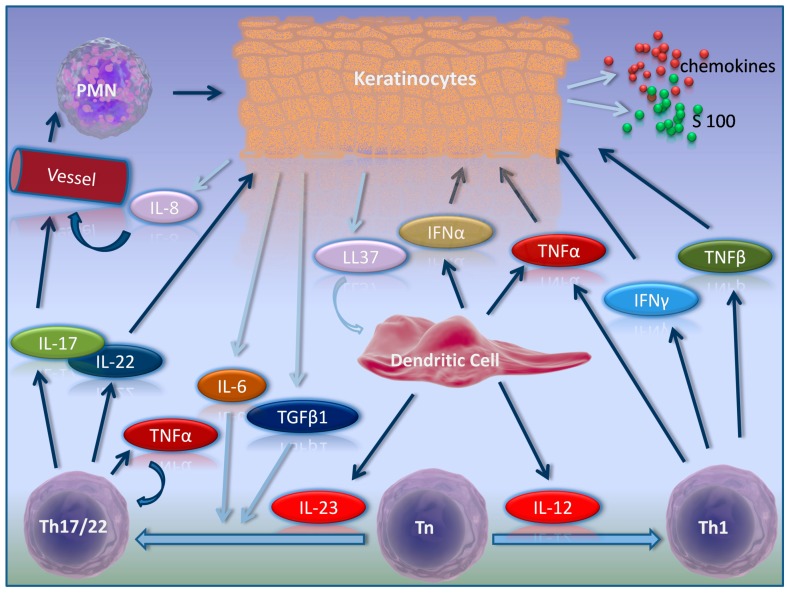
Cytokine network in psoriasis. IFNα  =  interferon-α, IFNγ  =  interferon-γ, IL-6 = interleukin-6, IL-8  =  interleukin-8, IL-12  =  interleukin-12, IL-17  =  interleukin-17, IL-22  =  interleukin-22, IL-23  =  interleukin 23, LL37 = cathelicidin, PMN  =  polymorphonuclears, S 100 = S 100 proteins, Th1  =  T helper 1, Th17  =  T helper 17, Th22 = T helper 22, TGFβ  =  transforming growth factor-beta, Tn  =  naïve T lymphocyte, TNFα  =  tumor necrosis factor-α, and TNFβ  =  tumor necrosis factor-β.

There are two commonly acknowledged phases for the pathogenesis of psoriasis: the initiation/triggering of the disease, and the maintenance of the pathological status [12]. In the early phase, DCs are activated and start producing inflammatory mediators. Plasmacytoid DC (pDCs) express Toll-like receptors (TLR)7 and TLR9 which normally recognize viral and microbial acids and do not respond to self-DNA [17]. Under certain conditions, which include triggering factors such as physical injury, keratinocytes produce excessive AMPs, such as β-defensins and LL-37. Damaged cells also produce self-nucleic acids, self-DNA and self-RNA. LL-37 binds self-DNA and forms complexes which are delivered in the early endocytic departments and which cannot be degraded and are able to activate TLR9 and TLR7, inducing IFN-α production and triggering pDC activation. On the other hand, self-RNA and LL-37 complexes stimulate myeloid DCs to mature after the production of TNF-α and IL-6 [12,18]. Once activated, DCs are transformed into mature antigen presenting cells and start producing cytokines like TNF-α, IL-23 and IL-12 and are therefore able to interact with T naive cells. IL-23, in association with IL-6 and TGF-β1, will determine the transformation of CD4+ naive cells to Th-17 which will produce IL-17, IL-22 and TNF-α. IL-23, in association with IL-6 and TNF-α, also promotes the production of Th-22 cells which secrete IL-22 and TNF-α [19]. All these mediators further maintain keratinocytes activation producing the now so-called auto-antigen, LL-37, proinflammatory cytokines (TNF-α, IL-1β, IL-6), chemokines and S100 proteins, propagating the chronic inflammation [20]. Altogether, these promote keratinocyte proliferation, production of AMPs and chemokines which promote neutrophil recruitment and sustain skin inflammation (Figure 1) [12,21,22,23,24].

## 3. Activators of Inflammation in Psoriasis

### 3.1. Cytokines

#### 3.1.1. IL-23

IL-23 is part of the IL-6/IL-12 family of heterodimeric cytokines and is composed of a p40 subunit, which is shared with IL-12, and a unique p19 subunit [12,25,26]. IL-23 is involved in the immune response against bacteria and fungi and is produced by several cells, including keratinocytes, dermal myeloid cells and macrophages [12,26]. The cytokine exerts its action through a receptor complex which is composed of the IL-23R subunit and the IL-12Rβ1 subunit, common with IL-12. IL-23 acts on cells expressing IL-23R, namely memory T cells, mast cells, macrophages, neutrophils, natural killer (NK) cells and keratinocytes [12,25]. The phosphorylation of Signal Transducer and Activator of Transcription (STAT) is required for signal transmission, STAT-3 being particularly important in psoriasis [25]. IL-23 has a paramount importance in maintaining the cytokine milieu required for the survival of Th-17 cells [27]. Naive T-cells do not express IL-23R and therefore cannot be directly activated by IL-23. In the presence of a combination of cytokines naive T-cells differentiate into Th-17 cells [27,28]. The model behind Th-17 differentiation is different in mice versus humans. In the murine model, IL-6 and TGF-β induce naive murine T-cells to differentiate into Th-17 cells and IL-21, IL-23, TNF-α and IL-1β amplify the development of those cells. In the human model, the optimal conditions for Th-17 differentiation are less clear and multiple combinations of cytokines have been described, including IL-1β and IL-6 or IL-23 and IL-1β. Moreover, in humans, the role of TGF-β in the differentiation of Th-17 cells is controversial [29].

Studies showed increased levels of IL-23, subunits p19 and p40 in psoriatic skin compared to non-lesioned skin [28]. Biological treatments targeting IL-23 are now intensely studied for the treatment of psoriasis and the preliminary results are promising [30].

#### 3.1.2. IL-1β

IL-1β is mainly produced by monocytes, macrophages, Langerhans cells and dendritic cells. It exerts its activity by binding to its receptor which is composed of the IL-1 receptor type 1 (IL-1R1) and IL-1 receptor accessory protein (IL-1RAcP). Activated IL-1R1 binds to the adaptor protein Myeloid differentiation primary response gene 88 and activates one or more Interleukin-1 receptor-associated kinases (IRAKs) [1]. Since IL-1β is a well-known initiator and effector for inflammation, several proteins involved in the production of this interleukin in psoriasis were investigated. CCN1 (cysteine-rich protein 61), a protein involved in inflammation, cell proliferation and angiogenesis, among others, was shown to be upregulated in psoriasis skin lesions and to promote keratinocyte proliferation via the α6β1/PI3K/Akt/NF-κB pathway, but was also shown to increase the production of IL-1β via the p38 MAPK signaling pathway. This suggests that CCN1 plays a role in the pathogenesis of psoriasis and in modulating inflammation in the disease [31]. In psoriasis, IL-1β maturation is mediated by ASC (Apoptosis-associated speck-like protein containing a caspase recruitment domain) -dependent inflammasome complexes, such as NLRP3 and AIM2, which activate caspase-1. However, NLRP-1, a subset of the NLR family inflammasomes, can further activate IL-1β by utilizing IL-17A promoted caspase-5, independently from ASC-dependent inflammasome. Therefore, caspsase-5 and NLRP-1 are potential targets for new psoriasis therapies [32].

#### 3.1.3. IL-17

IL-17A plays a leading role in the pathogenesis of psoriasis. It is a member of the IL-17 family which comprises six members, namely IL-17A, IL-17B, IL-17C, IL-17D, IL-17E and IL-17F. IL-17A and IL-17F are the most closely related and have overlapping biological functions. IL-17A is produced by Th17 cells, NK cells, γδT cells and innate lymphoid cells (ILCs), but also myeloid cells, B-cells, mast cells, neutrophils and macrophages. IL-17A and IL-17F signal through the heterodimeric receptor IL-17RA/IL-17RC which is located on keratinocytes, endothelial cells and fibroblasts [12,33,34,35,36]. IL-17A and IL-17F are involved in the protection against infections on epithelial and mucosal surfaces, especially staphylococcus and candida infections, and are key drivers in skin inflammation [33]. After binding to IL-17RA/IL-17RC, IL-17A determines the expression of AMPs like β-defensins, S100 proteins, LL37 and Lipocalin-2 (Lcn2), GM-CSF (Granulocyte-macrophage colony-stimulating factor), chemokines like CCL20, CXCL-1, CXCL-3, CXCL-5, CXCL-8, CCL-20, matrix metalloproteinases (MMP) and proinflammatory cytokines like IL-6, TNF-α and IL-1F9. CXCL-1, CXCL-3, CXCL-8 and AMP are involved in the recruitment of neutrophils at the infection site while CCL20 recruits Th-17 cells and ILC3 [12,33]. An aberrant production of IL-17A disrupts the appropriate immune responses and promotes the development of various inflammatory diseases, including psoriasis, rheumatoid arthritis, Crohn’s disease, etc. [35]. AMPs, cytokines and chemokines produced due to IL-17A have an important role in the formation of the psoriatic plaque [37].

Previous studies showed that IL-17 levels are increased in psoriatic patients [38,39] and the role of the interleukin in psoriasis is further supported by the favorable results obtained with biological agents targeting IL-17 [35,40,41].

Even though IL-17A and IL-17F are the most important of the IL-17 family members involved in the pathogenesis of psoriasis, IL-17E is also increased in keratinocytes from the psoriasis plaque and seems to play a proinflammatory role, as it is implicated in macrophage activation [42].

IL-17 has also been linked to cardiovascular disease and other inflammatory comorbidities. Therefore, targeting this interleukin might be associated with supplementary benefits for the psoriatic patient [23].

#### 3.1.4. IL-22

IL-22 is a member of the IL-10 family and plays an important role in the homeostasis of mucosa and barrier organs, as it has anti-bacterial, anti-fungal, anti-viral and anti-inflammatory activities. It is produced by Th-17, ILC3, mast cells, dermal γδ T cells, but also Tc-17 and Th-17 cells [43,44]. IL-22 receptors are made up of IL-22R1 and IL-10R2 subunits. IL-22R1 are restricted to non-hematopoietic cells. After IL-22 binds to the IL-22R complex, signal transmission is performed through the phosphorylation of STAT3 and the activation of the ERK1/2 pathway [45]. IL-22 production depends on IL-23 and transcription factors RORγt, but most importantly, AhR (aryl hydrocarbon receptor). AhR is a ligand transcription factor in Th-17 cells which is mandatory for the production of IL-22 by those cells. In psoriasis, the expression of IL-22 is increased and its effects are mostly directed towards regulating keratinocyte functions [12]. Therefore, IL-22 is involved in enhancing keratinocyte migration, increasing epidermal thickness by interfering with physiological desquamation, producing chemokines, AMPs, neutrophil chemoattractants and inducing production of MMPs [12,45]. Since IL-22 has a well-established role in the pathogenesis of psoriasis, this interleukin is a potential target for psoriasis treatments [45,46,47]. 

#### 3.1.5. IFN-γ

INF-γ is a type II interferon involved in innate and adaptive immunity against viral and intracellular bacterial infections [48]. Major sources of IFN-γ are activated Th-1 cells, CD8 T cells, NK cells and dendritic cells. The production of this cytokine is mostly controlled by IL-12 and IL-18 and macrophages, dendritic cells and naive T cells are the main responsive cells [1,49]. IFN-γ exerts its action through the IFN-γ receptor (IFNGR) which consists of two transmembrane chains: IFN-γ R1 and IFN-γ R2 [50] and primarily signals through the JAK-STAT pathway [49]. High levels of IFN-γ mRNA were identified in psoriasis lesions and psoriatic blood [51,52]. Kryczek et al. found that in humans, IFN-γ programs myeloid antigen presenting cells to produce IL-1 and IL-23 and to induce human IL-17+T cells [53]. In a study performed on 21 participants with psoriasis, the authors found a correlation between IFN-γ levels and disease severity measured by psoriasis area and severity index (PASI). Furthermore, IFN-γ was shown to be a prognostic factor for psoriasis [54]. However, in a study performed on 20 patients with psoriasis the authors found that treatment with a neutralizing anti-IFN-γ antibody had minimal efficacy and concluded that IFN-γ is not a major pathogenic cytokine in psoriasis lesions [55].

### 3.2. T-Cells

#### 3.2.1. Th-17 Cells

Th-17 cells are special populations of CD4+ T cells that produce IL-17, IL-22, IL-21, TNF-α and other cytokines, and express lineage specific transcription factor Retinoic acid receptor-Related Orphan receptor (RORC) [25,26,56,57]. The family of Th-17 cells includes several cell types, all of them expressing ROR-γt and IL-23R. Th-17 cells activated by IL-6, IL-1β and IL-23 trigger chronic inflammation and autoimmunity while TGF-β and IL-6 activated Th-17 cells are weakly pathogenic and are mostly involved in tissue integrity and defense [57,58,59]. 

#### 3.2.2. Th-22 Cells

Th-22 cells are a distinct subset of inflammatory CD4+ T cells with a unique expression profile and a novel functional spectrum. They produce IL-22, TNF-α, IL-13 and IL-26, but not IFN-γ or IL-17. The Th-22 phenotype can be promoted by TNF-α and IL-6 [60,61]. Additionally, they express chemokine receptors like CCR4 and CCR6 and are influenced by IL-23. Since the expression of IL-22 is the main feature of these cells, they were attributed the name Th-22 cells [45]. Increased levels of Th-22 [62], as well as Th-17, were identified in psoriasis vulgaris and psoriatic arthritis [63]. In a study performed on 60 patients with psoriasis and 30 healthy controls, the authors found significantly higher levels of IL-6, IL-20 and IL-22 in psoriatic patients than in the control group, the concentrations of IL-20 and IL-22 being positively correlated with disease severity measured with PASI and body surface area (BSA). The authors therefore concluded that the Th-22 response might contribute to the inflammatory disease in psoriasis [61]. Moreover, Cheuk et al. showed in a study published in 2014 that epidermal Th-22 and Tc-17 are retained in healed psoriasis and can produce cytokines involved in psoriasis pathogenesis, thus promoting disease recurrence in previously affected areas [64].

#### 3.2.3. Th-1 Cells

Th-1 cells are CD4+ T cells which produce IFN-γ, IL-2 and TNF and are the main carriers of cell-mediated immunity. Infections by intracellular bacteria and viruses are responsible for the production of Th-1 cells. IL-12 determines the differentiation of naive Th cells into Th-1 cells and enhances the production of IFN-γ [1,65]. While Th-17 cells play an important role in the initiation phase of the disease, Th-1 cells/IFN-γ-associated inflammation predominate in chronic plaques [66].

### 3.3. Other Molecules

#### RORC/RORγt

RORC is expressed on various immune cells and is a key factor in the differentiation of Th-17 cells. Animal studies have shown that RORγt (Retinoid-related orphan nuclear receptor gamma t, a ROR found in mice) deficient mice have decreased Th-17 cell differentiation associated with reduced inflammation [67]. In a study published in 2018, the authors determined the mRNA expression level of RORC in patients with psoriasis and found significantly higher gene expression of RORC in patients with psoriasis than in controls, thus concluding that Th-17 plays a role in the pathogenesis of the disease [56]. RORγt antagonists and inverse agonists are now being tested for the treatment of psoriasis [68].

## 4. Regulatory Axis in Psoriasis

### 4.1. Treg Cells

T regulatory (Treg) cells, more specifically the abnormalities in the Th17/Treg balance, were also shown to be key players in the pathogenesis of psoriasis [69]. Treg cells are a heterogenous group of T lymphocytes responsible for suppressing an excessive or autoreactive immune response, thus playing an important role in immunological tolerance [69,70]. Several regulatory cells populations have been described in humans, including regulatory B cells, NK-T cells, myeloid derived suppressor cells and CD8+ regulatory T cells, the most important however being a subset of T helper (CD4+) regulatory cells. The main phenotypic characteristics of Treg cells are the high expression of the CD25 receptor, the expression of the transcription factor forkhead box P3 (FOXP3) and the production of IL-10 and TGF-β [12,70]. Treg cells interact with other cells by producing suppressing cytokines like IL-10, IL-35 or TGF-β, by releasing granzyme B or perforin which have a direct cytotoxic action or through cell receptors [69]. Naive Treg cells can differentiate into Th1-Treg, Th2-Treg, Th17-Treg, Fat-Treg and Tfr-Treg which suppress Th-1, Th-2, Th-17, adipocytes and T-cells in the germinal centers of lymphoid tissue [70]. When the promoters of IL-10, IL-35, TGF-β1, CTLA4 (T-lymphocyte–associated antigen 4) and CD25 genes are activated and the promoters of IL-2, IL-4 and INF-γ are blocked, the produced Treg cells are suppressive [71]. FOXP3 mutations, which result in the absence or an inadequate number of Treg cells, manifests as IPEX syndrome: immune dysregulation, polyendocrinopathy, enteropathy and X-linked syndrome [70,71].

Abnormalities in Treg cells have been associated with inflammation in psoriasis. Therefore, by producing IL-10, Treg cells downregulate the expression of proinflammatory cytokines, chemokines and adhesion molecules and decrease inflammation [12]. A study performed on patients with plaque and guttate psoriasis found higher levels of FOXP3 positive Treg cells in skin lesions and peripheral blood of patients with plaque type psoriasis, the levels being positively correlated with disease severity [72]. Other authors also identified higher levels of FOXP3 positive Treg cells and Th-17 in the psoriatic lesions and the peripheral blood, thus indicating a possible role in the pathogenesis of psoriasis [73,74]. However, some authors found lower levels of Treg cells not only in the peripheral blood of psoriatic patients [75], but also in skin samples [70]. Since the presence of Treg cells in the psoriatic plaque was not associated with a decrease in inflammation, several authors investigated those cells. Soler et al. showed in a study published in 2013 that psoriatic Treg cells are numerically, functionally and chemotactically deficient and are therefore unable to restrain inflammation [76]. Moreover, it has been shown that under proinflammatory conditions, Treg cells can differentiate into Th-17 cells. In the presence of IL-6 and TGF-β, naive T cells upregulate both FOXP3 and RORγt. Thus, Treg and Th-17 subtypes compete for the same T cell precursor which upregulates FOXP3 and RORγt depending on the cytokine repertoire from the psoriatic milieu. Considerably increased in psoriasis, IL-23 is a pro-inflammatory cytokine attributed to regulate Treg cells via STAT3 pathway activation, thus disturbing Treg cell function, recognized as a hallmark of psoriasis [69]. Bovenshen et al. therefore showed that FOXP3 positive Treg cells from psoriasis lesions can differentiate into a strong proinflammatory triple positive IL-17A+/Foxp3+/CD4+ Th-17 which perpetuates the inflammatory process [77]. 

Micro-RNAs (miRs) are a group of small, endogenous, non-coding RNAs, composed of approximately 21–23 nucleotides, which negatively regulate gene expression at the posttranscriptional level. More than 2500 miRs have been identified in humans. Of those, miR-210 is significantly increased in the peripheral blood mononuclear cells and skin lesions of patients with psoriasis [78,79]. In a study published in 2014, the authors reported that microRNA-210 is overexpressed in CD4+ T cells from psoriatic patients and that it inhibits expression of FOXP3, thus impairing the immunosuppressive function of Treg cells. Moreover, miR-210 inhibition reverses the immune dysfunction [80]. Regulation of miR-210 expression in psoriasis has direct consequences on immune dysfunction exerted by Tregs. Thus, in a recent study performed on mice, Wu et al. showed that miR-210 ablation and its inhibition by antagomir-210 blocks the immune imbalance and the development of the psoriatic plaque [79]. A specific role in miR-210 effect is attributed to hypoxia-inducible factor-1α (HIF-1α) a transcription factor known to modulate genes associated to hypoxic milieus [81]. In addition, in psoriasis HIF1α is known to induce miR-210 overexpression at CD4+ T cells level mediated by TGF-β and IL-23. HIF1α act in an epigenetic manner by causing hyperacetylation of histone H3 in the miR-210 gene promoter revealing a potentially upstream regulatory mechanism of miR-210 overexpression. These data endorse miR-210 for a proinflammatory role in inducing immune imbalance and skin lesion presence in psoriasis [79].

### 4.2. TGFβ

TGFβ is a multipotent growth factor involved in maintaining immune homeostasis. It inhibits the activity of macrophages and neutrophils, promotes angiogenesis and the proliferation of fibroblasts and regulates T cells subpopulations [69]. Three isoforms are currently recognized, respectively TGFβ-1, TGFβ-2 and TGFβ-3, which bind to their receptors, TGFβRI, TGFβRII and TGFβRIII. Of those, TGFβ1 is predominantly found in the skin [82]. Studies showed that elevated serum levels of TGF-β1 are found in psoriatic patients and that those levels are correlated with disease severity [83,84]. TGFβ-1 was considered an anti-inflammatory cytokine. However, its overexpression in keratinocytes was shown to induce skin inflammation and the development of psoriasis-like lesions via a Smad-dependent mechanism [82,85]. In patients with psoriasis, TGFβ-1 induces the generation of FOXP3 positive Treg cells in the absence of IL-6 and the production of Th-17 cells in the presence of IL-6 [86]. While the exact role of TGF-β in the pathogenesis of psoriasis is not completely understood, data available so far suggests that it might be a good biomarker for the severity of psoriasis and treatments targeting Smad3 might be associated with favorable results [7].

### 4.3. IL-10

IL-10 is probably one of the most potent anti-inflammatory cytokines and has an important role in immune mediation. Its part in modulating the immune response to microbial flora raised the suspicion that it is involved in the pathogenesis of various inflammatory diseases, including psoriasis [87]. Macrophages are the most important source of IL-10, but it is also secreted by B cells, T cells, mast cells, dendritic cells, keratinocytes, eosinophils and NK cells, among others [87,88]. Moreover, there are recent studies that link psoriasis onset with mutations in the promoter region of the *IL-10* gene [89]. IL-10 performs its regulatory actions through the modulation of antigen presentation in dendritic cells, suppression of T cell activity and stimulation of B cell differentiation [87,88]. Studies performed in patients with psoriasis showed that the levels of IL-10 are decreased in the patients’ serum [90,91]. In a study performed on peripheral blood B regulatory cells (Bregs) from 60 patients with psoriatic arthritis, 50 patients with psoriasis and 23 healthy controls, the authors found that IL-10 producing Bregs were decreased in patients with psoriasis and psoriatic arthritis and that they were inversely correlated with disease severity [92]. Various psoriasis treatments have been associated with an increase in the levels of IL-10. Zanin-Zhorov et al. showed that the oral administration of KD025, a selective inhibitor of Rho-associated kinase (ROCK)2—a serine/threonine kinase protein involved in regulation of autoimmunity—leads to a decrease in disease severity measured by PASI, a decrease in pro-inflammatory cytokines IL-17 and IL-23 and an increase in IL-10 levels after 10 weeks of treatment [93]. *Cyanobacterium aponinum*, a member of the microbial ecosystem of the Blue Lagoon in Iceland, was also shown to have beneficial effects in psoriasis. In a study published in 2015, the authors found that exopolysaccharides (EPSs) secreted by *C. aponinum* determines the maturation of dendritic cells, increased the levels of IL-10 and the frequency of FoxP3(+)IL-10(+) T cells and decreased the IL-17(+)RORγt(+)/FoxP3(+)IL-10(+) ratio. The authors therefore concluded that bathing in the Blue Lagoon could be advantageous for psoriatic patients [94]. All this data supports the role of IL-10 in the pathogenesis of psoriasis and supports the idea that targeting IL-10 might be useful in psoriasis. Further data is however required.

## 5. Additional Inflammatory Pathways in Psoriasis

There are several recent pro-inflammatory pathways that were linked to psoriasis pathogenesis. ACKR2 (Atypical chemokine receptor 2), previously known as the chemokine-scavenging receptor D6, is a scavenger receptor for CC chemokines that has been associated with various inflammatory diseases, including psoriasis. In the skin, ACKR2 is expressed by keratinocytes and dermal lymphatic endothelial cells. Unlike other chemokine receptors, ACKR2 are unable to mount typical signaling responses to chemokines, but instead internalize and degrade inflammatory chemokines [95]. Singh et al. observed that this receptor is markedly expressed in uninvolved psoriatic skin and that inflammatory, but non-functional, CC chemokines are also increased in uninvolved skin. The authors therefore concluded that ACKR2 plays a part in suppressing chemokine-driven inflammatory responses [96]. Shams et al. managed to link altered ACKR2 expression in psoriasis to miR-146 and miR-10b, two microRNAs that directly bind ACKR2 3′-untranslated region and decrease the expression of ACKR2 transcripts in keratinocytes and lymphatic endothelial cells. Furthermore, the authors showed that cell trauma, a well-known trigger for psoriasis, also leads to decreased expression of ACKR2 [97]. Animal studies found that mild inflammation and IFN-γ administration are able to increase ACKR2 expression and restrict inflammation. ACKR2 induction might therefore be a promising therapeutic strategy in psoriasis [98]. 

Even though psoriasis is considered a T cell mediated disease, some authors investigated the potential role of B cells in the pathogenesis of psoriasis. In a study published in 2016, the authors reported higher levels of CD19+ B cells in the peripheral blood of psoriatic patients than in healthy controls. Moreover, CD19+ B cells ratios were positively correlated with disease severity and the authors therefore concluded that B cells might play a role in different pathological stages of psoriasis [99]. B regulatory cells are a subset of B cells that can negatively regulate immune responses. In a study performed on mice, the authors showed that the skin inflammation induced by imiquimod was more severe in CD19−/− mice than in wildtype mice and that regulatory B cells can suppress the psoriasis-like inflammation [100]. Depletion of B cells with rituximab was associated with the development of a psoriasis-like eruption in a patient treated for autoimmune lymphoproliferative syndrome type III [101]. On the other hand, in a study published in 2018 by Thomas et al., the authors concluded that B cells alterations are only an epiphenomenal finding in psoriasis [102]. Further studies are therefore needed to support the role of B cells in psoriasis disease specific inflammation.

Beside those, panoply of molecules and cells with a potential role in the pathogenesis of psoriasis has recently been investigated. We will further discuss some of the most recent findings in both humans and animal models (Table 1). 

Harden et al. explored the expression of the tryptophan metabolism enzyme L-kynureninase (KYNU) in psoriatic human skin, normal human skin, blood cells and primary cells and found KYNU+ cells in psoriatic lesional cells, their expression being positively correlated with disease activity [103]. CD5+ dendritic cells, which can activate cytotoxic T cells and Th22 cells, were also found in higher levels in skin biopsies from patients with psoriasis than in biopsies from adjacent uninvolved skin, thus suggesting their role in the disease [104]. Buerger et al. showed in a study published in 2017 that mTOR signaling, which is normally deactivated when keratinocytes switch from proliferation to terminal differentiation, under inflammatory conditions have an aberrant activity leading to enhanced proliferation [105]. Nuclear receptor interacting protein 1 (NRIP1), a co-regulator for numerous nuclear receptors, was found to be overexpressed in psoriatic lesions and in peripheral blood mononuclear cells of patients with psoriasis. Moreover, animal studies showed that imiquimod-induced inflammation was delayed in knockout NRIP1 mice. The authors suggested that NRIP1 could induce abnormal proliferation and apoptosis of keratinocytes through direct interaction with Re1A/p65 [106].

In a mouse preclinical model, Andrianne et al. studied the expression of tristetraprolin (TTP), an RNA-binding protein encoded by Zfp36 gene which regulates the mRNA stability of some cytokines, in keratinocytes and explored its role in the imiquimod-induced psoriasis model. The authors found that TTP deficiency is associated with systemic inflammation, skin lesions and psoriasis related arthritis [107]. Deficiency of VISTA (V-domain Immunoglobulin Suppressor of T cell Activation), an inhibitory immune checkpoint protein which suppresses CD4+ and CD8+ T cell activation, was also associated with exacerbated psoriasiform inflammation due to hyperactivation of Erk1/2 and Jnk1/2 and increased production of IL-23 [108]. In an imiquimod-induced psoriatic mouse model, Surcel et al. have shown that in both peripheral blood and secondary lymphoid organs disease development is associated with a significantly increased T-CD8a+ and NK1.1+ cell percentages while decreased T-CD4+ and B lymphocyte percentages [109]. Furthermore, the deficiency of TWEAK, a molecule of the TNF superfamily [110], the overexpression of Glucocorticoid-induced Leucine Zipper (GILZ) [111], prokineticin 2 (PK2) [112], upregulation of ANGPTL6 [113], Human β-Defensin 3 and Murine β-Defensin [114] were shown to have an inflammatory effect while the natural plant antimicrobial solution (PAM) [115], the endoribonuclease MCPIP1 [33], the flavone Luteolin-7-glucoside (LUT-7G) [46], Heme oxygenase-1 (HO-1) [116], the flavonoid Astilbin [117], Paeoniflorin (PF) and Paeonol (PN)-ingredients from plants used in Traditional Chinese Medicine [118,119], superoxide dismutase (SOD3)-transduced Mesenchymal Stem Cells [120] have an anti-inflammatory effect.

## 6. Conclusions

Psoriasis is an immune-mediated, inflammatory, polygenic skin disorder with a great impact on patients’ quality of life. Despite being one of the most studied dermatological afflictions, the exact pathogenic mechanism leading to disease associated inflammation is still not completely understood. There is now enough evidence to support the role of IL-23, IL-17, IL-22, Th-17 cells, Th-22 cells, and TGF-β1 in the pathogenesis of the disease. Moreover, several inflammatory and anti-inflammatory molecules are currently being investigated, some of them showing promising results. One should, however, keep in mind that many of those molecules are involved in normal physiological processes and/or in fighting viral, bacterial or fungal infections, among others. Inhibiting some of those molecules might therefore be associated with adverse events. The development of novel, efficient topical treatments could potentially help reduce the frequency of unwanted reactions. 

Identifying new pieces in the puzzle represented by the cells and cytokines involved in the pathogenesis of psoriasis might help identify new biomarkers for disease diagnosis and assessment and new, potentially better, treatments.

## Figures and Tables

**Table 1:** Overview of the cell/molecule that induces a particular effect and the subsequent model used to demonstrate the effect.

Biological Effect	Cell/Molecule/Pathway	Model Used to Demonstrate the Effect	Reference
Cell-Type Involvement/Effects			
Inflammation	CD5+ dendritic cell by inducing cytotoxic T cells and Th22 cells	Skin samples from psoriatic patients and healthy controls	[104]
Inflammation	Significantly increased peripheral T-CD8a+ lymphocyte and NK1.1+ cell percentages, decreased peripheral T-CD4+ and B lymphocyte percentages.	Samples from imiquimod experimental psoriasis mouse model	[109]
Cytokines/Chemokines-Type Effects			
Inflammation	TWEAK (TNF superfamily molecule)	TWEAK-deficient mice bred on the C57BL/6 background; Fn14-deficient mice bred on BALB/c background	[110]
Anti-inflammatory	MCPIP1/Regnase-1 via restriction of IL-17A and IL-17C signaling	Skin biopsies from psoriatic patients; *Zc3h12a*^−/−^ mice; *Il17ra*^−/−^ mice; *Il17a*^−/−^ mice.	[33]
Bioactive Molecules-Type Effects			
Inflammation	Upregulated L-kynureninase (KYNU)	Skin and blood samples from psoriatic patients and healthy controls	[103]
Inflammation	Nuclear receptor interacting protein 1 (NRIP1) via the regulation of RelA/p65	Skin and blood samples from psoriatic and healthy patients; HaCaT cells; C57BL/6J (B6) and *Nrip1*^−/−^ mice	[106]
Inflammation	aberrant mTORC1 signaling	Spontaneously immortalized human keratinocyte cell line (HaCaT); NHK (normal human keratinocytes).	[105]
Inflammation	Tristetraprolin (TTP) deficiency	Zfp36-deficient mice (Zfp36^−/−^); LoxP-flanked Zfp36 mice (Zfp36^fl/fl^); LysM-Cre mice; CD11c-Cre mice; K14-Cre mice; *Zfp36*^ΔEP^*Tnf*^ΔEP^ mice	[107]
Inflammation	VISTA (V-domain Immunoglobulin Suppressor of T cell Activation) deficiency via hyperactivation of Erk1/2 and Jnk1/2.	C57BL/6 mice; *Vsir*^−/−^ mice	[108]
Inflammation	Overexpression of GILZ (Glucocorticoid-induced Leucine Zipper) via activation of TGF-β1	GILZ-Tg (transgenic)mice; GILZ-Wt	[111]
Inflammation	High expression of PK2 (prokineticin 2) induces production of IL-1 in macrophages	K14-VEGF transgenic mice; Kunming mice; C57BL/6 mice	[112]
Inflammation	Upregulation of epidermal ANGPTL6 promotes hyperproliferation of keratinocytes	K14-Angptl6 Tg mice; skin biopsies from psoriasis patients.	[113]
Inflammation	Human β-Defensin 3 and Murine β-Defensin 14 via Langerhans cell activation	Skin biopsies from psoriatic patients; C57BL/6 mice.	[114]
Anti-inflammatory	PAM (plant antimicrobial solution) via inhibition of inflammatory NF-κB signaling pathway	HaCaT cells; Female BALB/c mice	[115]
Anti-inflammatory	Luteolin-7-glucoside via inhibition of IL-22/STAT3 pathway	HEKn cells (Human Epidermal Keratinocytes, neonatal); C57BL/6 mice	[46]
Anti-inflammatory	Astilbin inhibits Th17 cell differentiation via Jak3/Stat3 signaling pathway	BALB/c mice	[117]
Anti-inflammatory	Heme oxygenase-1 (HO-1) by negative regulation of STAT3 signaling	HaCaT cells; biopsies from psoriatic patients; BALB/c mice	[116]
Anti-inflammatory	Paeoniflorin by regulating Th17 cell response via phosphorylation of STAT3	BALB/c mice; C57BL/6 mice	[118]
Anti-inflammatory	Paeonol by inhibiting the maturation and activation of DC via the TLR7/8 signaling pathway	BALB/c mice	[119]
Anti-inflammatory	Superoxide dismutase (SOD3)-transduced MSCs (Mesenchymal Stem Cells) via inhibition of signaling pathways toll-like receptor-7, nuclear factor-kappa B, p38 mitogen-activated kinase, and Janus kinase–signal transducer and activator of transcription	C57BL/6 mice	[120]
